# Learner-Centered Experience-Based Medical Education in an AI-Driven Society: A Literature Review

**DOI:** 10.7759/cureus.46883

**Published:** 2023-10-12

**Authors:** Nobuyasu Komasawa, Masanao Yokohira

**Affiliations:** 1 Community Medicine Education Promotion Office, Faculty of Medicine, Kagawa University, Takamatsu, JPN; 2 Department of Medical Education, Kagawa University, Takamatsu, JPN

**Keywords:** literacy, learning, healthcare, artificial intelligence, experience

## Abstract

This review proposes and explores the significance of "experience-based medical education" (EXPBME) in the context of an artificial intelligence (AI)-driven society. The rapid advancements in AI, particularly driven by deep learning, have revolutionized medical practices by replicating human cognitive functions, such as image analysis and data interpretation, significantly enhancing efficiency and precision across medical settings. The integration of AI into healthcare presents substantial potential, ranging from precise diagnostics to streamlined data management. However, non-technical skills, such as situational awareness on recognizing AI's fallibility or inherent risks, are critical for future healthcare professionals. EXPBME in a clinical or simulation environment plays a vital role, allowing medical practitioners to navigate AI failures through sufficient reflections. As AI continues to evolve, aligning educational frameworks to nurture these fundamental non-technical skills is paramount to adequately prepare healthcare professionals. Learner-centered EXPBME, combined with the AI literacy acquirement, stands as a key pillar in shaping the future of medical education.

## Introduction and background

In recent years, the remarkable advancements in the field of artificial intelligence (AI) have led to the emergence of third-generation AI, predominantly powered by deep learning. This cutting-edge form of AI is revolutionizing numerous aspects of the medical domain, showcasing immense potential for transformative applications. One of the key strengths of this AI paradigm is its ability to replicate and utilize intellectual processes akin to that of humans, thereby greatly augmenting the efficiency and effectiveness of various medical settings [1].

First-generation AI, often referred to as "symbolic AI" or "good old-fashioned AI," focused on rule-based systems and symbolic reasoning. Researchers programmed explicit rules and logic to represent knowledge and solve problems. These systems were rule-bound and lacked learning capabilities. Examples include expert systems and early natural language processing. Second-generation AI witnessed a shift toward machine learning and statistical methods. Rather than relying solely on handcrafted rules, AI systems were designed to learn patterns and make predictions from data. Techniques, such as neural networks, decision trees, support vector machines, and clustering algorithms, became prevalent. This era saw significant advancements in supervised learning, unsupervised learning, and reinforcement learning [2].

In contrast to first- or second-generation AI, third-generation AI, through its sophisticated algorithms and neural networks, can engage in complex tasks comparable to human cognitive functions. Third-generation AI includes intricate processes, such as pattern recognition, data analysis, discovery of hidden patterns and correlations within vast datasets, and learning from previous experiences. By effectively mimicking these higher-order cognitive functions, AI can significantly contribute to medical research, diagnosis, and treatment planning [3]. An illustrative example is AI-powered diagnostic systems that analyze medical imaging, such as X-rays or MRI scans, to detect anomalies and assist healthcare professionals in making accurate diagnoses [4].

Moreover, the integration of third-generation AI into healthcare systems has brought about a substantial transformation in the management and analysis of medical information [5]. AI systems are capable of swiftly processing enormous volumes of health data, extracting valuable insights, and organizing the information in a structured manner. This streamlined data management not only enhances the efficiency of clinical operations but also lays the foundation for evidence-based medical practices. For instance, AI-driven electronic health record systems can analyze and organize patient data, providing comprehensive summaries to healthcare practitioners, thus saving valuable time and ensuring a more informed decision-making process [6].

While AI technology has dramatically evolved, aligning educational frameworks to nurture these fundamental non-technical skills associated with AI's fallibility or inherent risks is paramount to adequately prepare healthcare professionals. To cultivate these non-technical skills, active reflection learners' own experience is warranted. In other words, learner-centered approach on experience is essential. 

In this review, we propose the significance of learner-centered "experience-based medical education" (EXPBME) and the acquisition of AI literacy as essential components in shaping the future of medical education.

## Review

Transformative potential of third-generation AI in healthcare

The ongoing advancements in third-generation AI are poised to bring about a paradigm shift in healthcare, augmenting medical practices, empowering healthcare professionals, and ultimately enhancing the quality of care provided to patients [4]. The integration of AI into the medical landscape holds the promise of alleviating the burden on healthcare professionals, particularly physicians [1]. By automating routine and time-consuming tasks, AI allows physicians to redirect their focus and expertise toward more personalized and patient-centric care. For example, AI-powered algorithms can help in automating administrative tasks, appointment scheduling, and prescription management, allowing physicians to dedicate more time to direct patient interactions, empathetic communication, and devising tailored treatment plans based on individual patient needs [7].

Looking forward, as AI technology continues to evolve and mature, it is poised to revolutionize the entire healthcare ecosystem. AI accelerates drug discovery by analyzing vast datasets to predict drug behaviors, identify potential compounds, and optimize drug properties. This expedites the research and development process, saving time and resources. AI tailors medical treatments to individuals by analyzing genetic, clinical, and lifestyle data. This enables customized therapies and medication plans, optimizing outcomes based on a person's unique characteristics and health history. AI enhances telemedicine by providing diagnostic support, remote monitoring, and personalized health recommendations. It improves accessibility to healthcare services, especially in remote or underserved areas, by leveraging technology for virtual consultations and efficient healthcare delivery. This evolution is expected to progressively shift the traditional workflows of physicians, placing a stronger emphasis on patient-centered care and multidisciplinary collaboration [8]. The synergy between AI technology and medical professionals is anticipated to drive unprecedented advancements in clinical decision-making and, consequently, lead to improved patient outcomes [4].

The collaborative integration of AI with the medical field holds immense promise, paving the way for a future where healthcare is more personalized, efficient, and effective [9].

Ethical and educational imperatives in AI integration for enhanced healthcare

Striking a harmonious balance between technological advancements and ethical integrity is central to realizing the full potential of AI integration in medical practice for the betterment of healthcare outcomes and patient welfare. The rapid proliferation of AI applications within the realms of medical and healthcare necessitates a heightened awareness among medical educators on a global scale regarding AI and the multifaceted challenges it entails [10,11]. This imperative stems from the transformative potential AI holds in reshaping medical education, particularly in the domains of precise diagnosis and surgical procedural support. 

However, in this promising landscape of AI-driven advancements in healthcare, ethical and legal concerns loom large. Issues relating to data privacy, algorithmic bias, accountability, and the potential for overreliance on AI require careful deliberation and the establishment of appropriate regulatory frameworks [12]. Medical educators and institutions have an important role in addressing these concerns. They need to ensure that future healthcare professionals understand the ethical responsibilities and legal implications associated with integrating AI into healthcare [13,14].

To ensure that medical students are well prepared for the evolving healthcare landscape, academic institutions are proactively reshaping their curricula to encompass AI and data science literacy [15]. The objective is to equip aspiring healthcare professionals with the requisite knowledge and skills to harness AI effectively, discern its underlying principles, and navigate associated risks. This entails not only instructing students on how to use AI tools proficiently but also instilling a deep comprehension of AI's intricacies and its potential impact on healthcare [16].

A critical aspect of this educational paradigm involves integrating competency development related to AI and its legal and ethical issues into medical education curricula [17]. It is essential to cultivate problem-solving skills pertinent to the AI era in medical students, which encourages a proactive approach to addressing the complex and evolving landscape of healthcare augmented by AI. Medical students must be nurtured to think critically and ethically, understanding the optimal application of AI in healthcare while upholding the highest standards of patient care and ethical conduct [18].

In sum, as AI applications continue to gain prominence within the medical and healthcare domains, a proactive and informed approach to medical education becomes paramount. The evolution of educational frameworks to encompass AI and data science literacy, coupled with a strong emphasis on ethical considerations and competency development, will play a pivotal role in preparing future healthcare professionals to navigate and capitalize on the dynamic healthcare landscape effectively [19].

Caution to excessive digital dependency in medical education in the AI era

Digital transfer in the medical environment refers to the application of information and communication technologies (ICTs) driven by AI to support health and healthcare, including electronic medical records, videoconferencing technology, and wearable devices, such as mobile applications or virtual/augmented reality [20,21]. These technologies support clinical and administrative processes, enhance access to medical services, and empower patients to monitor and manage their health [22,23]. In addition, digital technology has significantly impacted medical education, transitioning from traditional handwritten learning to digital methods, influencing learning styles and competencies of medical students [24,25]. AI-driven ICT advancements have not only revolutionized the way medical information is accessed and disseminated but have also significantly impacted medical education and training [26,27].

The onset of the COVID-19 pandemic served as a catalyst, accelerating the digitization of medical education through the widespread adoption of remote classes and virtual training platforms [28]. This shift, while essential for ensuring continuity in education during challenging times, has raised valid concerns regarding the potential impediments posed by heavy reliance on digital devices. There is a growing apprehension that an overreliance on digital tools might hinder experiential-based learning and inhibit a deep and nuanced understanding of clinical medicine among students [29].

Extensive research has shed light on the elevated levels of digital dependency observed among medical students [30]. While digital devices undeniably facilitate clinical skill acquisition by providing an extensive repository of image-based information, experiential and hands-on training remains fundamentally indispensable for the development of pertinent competencies. It is incumbent upon medical educators to be acutely aware of the potential risks associated with excessive use of digital devices and the resultant digital dependency. Consequently, there is a pressing need to adapt and evolve educational approaches to strike a delicate balance that maximizes the benefits while minimizing the drawbacks inherent by cultivating AI literacy. This entails a thoughtful integration of technology into the curriculum, promoting active and engaged learning and fostering critical thinking skills necessary for future healthcare professionals.

A comprehensive approach could involve using interactive educational platforms for the practical application of theoretical knowledge. Combining AI resources with hands-on experiences through blended learning creates a holistic learning environment. The AI-driven digital age offers transformative potential for medical education and practice. It is vital to balance digital advancements with experiential learning to nurture healthcare professionals proficient in technology and clinical skills, optimizing healthcare outcomes in today's digital landscape.

Essential role of non-technical skills derived from experience in the AI-driven society

Both medical educators and learners should remain cognizant of the potential risks associated with an overreliance on AI-generated information and emphasize the continued importance of developing non-technical skills through experiential learning. Balancing the integration of AI-driven advancements with the essential cultivation of non-technical skills will be fundamental in preparing healthcare professionals to deliver safe, efficient, and compassionate care in the ever-evolving AI-dominated medical landscape. In the rapidly evolving landscape of the AI era, the significance of both technical and non-technical skills in medical practice cannot be overstated, as they play pivotal roles in ensuring optimal clinical outcomes and fostering advancements in patient care and medical safety [31,32]. Technical skills, often emphasized and honed through traditional medical education, are imperative for clinical proficiency. Equally vital, however, are the non-technical skills, which encompass a spectrum of cognitive resources enhancing task performance efficiency and encompass domains, such as situational awareness, decision-making, effective communication, and stress management [33,34].

Here, we propose the idea of EXPBME, which is based on various reflections based on experiences. EXPBME through clinical or simulation experience stands as an efficacious instructional method in cultivating both technical prowess and these crucial non-technical crisis management skills across diverse medical settings [35]. EXPBME is based on experiential learning theory in which reflection leading to new idea or skills is essential [36,37].

However, as we transition further into the AI era, a new layer of complexity is added to the acquisition of non-technical skills. Medical practitioners must now not only grapple with assimilating this vast trove of AI-generated data but also maintain their focus on developing the nuanced non-technical skills that are equally critical for effective patient care.

Significance of learner-centered EXPBME in the AI-driven society

**Figure 1:**
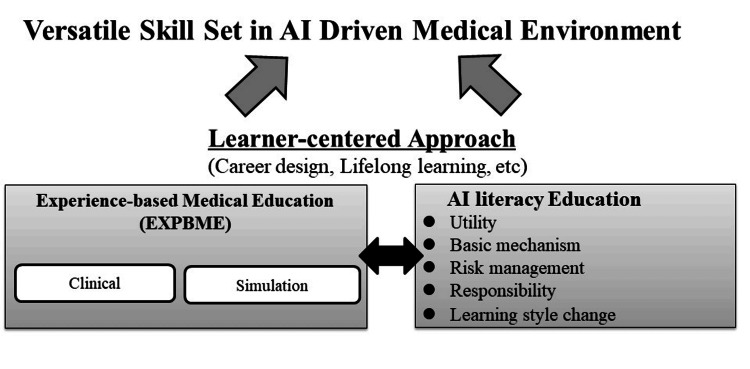
Learner-centered experience-based medical education (EXPBME) and AI literacy. This figure is the author's own creation.

The constantly changing field of medicine, driven by technology and breakthroughs in research, requires doctors to keep learning and improving their skills continuously [38]. Traditional medical school education, while providing a strong foundational understanding, falls short in meeting the burgeoning demands and complexity of modern healthcare. Thus, there is an imperative need for a paradigm shift toward a model of continuous career development and lifelong learning for medical professionals. To develop this competency on reflecting their own learning style, the concept of learner-centered EXPBME is warranted. In other words, deep and sufficient reflections based on experiences play an essential role for active learning even in the AI-driven society. The idea of EXPBME is compatible to outcome-based education (OBE) because these two ideas are both based on experiences (Figure 1).

Medical students must embrace the concept of lifelong learning and commence career design strategies early in their educational journey, preferably during their initial clinical training period following the attainment of their M.D. degree. Learner-centered EXPBME, such as early exposure to diverse clinical settings or mentored research experiences, plays a crucial role in shaping their career trajectories [39]. These experiences not only enrich their understanding of various specialties but also empower them to make informed decisions about their career paths, fostering a proactive approach to lifelong education and professional growth [40]. This guidance ensures that medical students are well informed about the multitude of options available to them, allowing them to align their interests, skills, and aspirations with a fulfilling and impactful career.

From the viewpoint of learner-centered EXPBME, incorporating professional development and career-oriented modules into the medical curriculum can further enhance students' readiness to design their careers effectively [40]. These modules can cover a spectrum of topics, including leadership skills, effective communication, interdisciplinary collaboration, and the ethical dimensions of modern healthcare. By integrating such elements into the education framework, medical schools can nurture well-rounded professionals equipped not only with clinical competence but also with the vital non-technical skills essential for success in today's AI-driven healthcare landscape. To meet the evolving demands of the medical field, medical education must transcend traditional boundaries. Early career design, lifelong learning, and comprehensive career counseling are pivotal components in empowering medical students to proactively shape their professional journeys and active learning attitudes. In addition, integrating professional development modules into the curriculum ensures that future medical doctors are not only proficient clinicians but also adept at navigating the complex and multifaceted landscape of modern medicine. Through these learner-centered strategies, we can cultivate a generation of healthcare professionals primed to make enduring and meaningful contributions to the ever-advancing realm of healthcare.

Significance of learner-centered EXPBME for acquiring non-technical skills regarding AI literacy

Experience-based learning, particularly through simulation exercises employing a rescue method, emerges as a cornerstone in equipping medical professionals with the indispensable competencies needed to mitigate and manage AI errors in real time. Emphasizing learner-centered EXPBME is crucial for developing non-technical AI skills in the medical field. It is rooted in core principles, preparing learners for AI's evolving role in healthcare education. In the AI era, EXPBME gains central importance in shaping medical education.

The ongoing transformation within healthcare, driven by the integration of AI and its allied technologies, necessitates a parallel evolution in medical education. Medical learners must develop advanced research skills that enable them to delve deeply into the intricate world of AI, not merely understanding the algorithms and mechanisms that power it but also critically evaluating and validating the outcomes that AI-powered systems produce [41]. 

While AI is precise and efficient, it is crucial to acknowledge its fallibility and the potential for errors. Healthcare professionals bear the responsibility for AI-driven diagnoses or suggestions, highlighting the need for human oversight. Understanding the complex deep learning mechanisms of AI can be challenging, underscoring the importance of non-technical skills in detecting AI failures. This underscores the necessity of integrating experiential learning methodologies, designed to simulate AI failures and immerse learners in situations that prompt swift and effective responses.

Learners must not only develop AI literacy but also acquire a high level of competency in non-technical skills through practical experiences. These skills empower them to identify errors or mistakes made by AI effectively. This, in turn, enhances patient safety and ultimately leads to the delivery of optimal healthcare outcomes in an AI-powered medical landscape that is in a constant state of evolution.

Further roles of learner-centered EXPBME in the AI era

In the evolving landscape of healthcare technology, the role of learner-centered EXPBME transcends its foundational principles, taking on a multifaceted significance in the AI era. Medical education programs, deeply rooted in learner-centered approaches, can elevate AI literacy development by replicating a spectrum of scenarios where AI systems may provide incorrect diagnoses or treatment recommendations. Through hands-on experiences in managing such diverse situations, medical learners not only cultivate a keen awareness of AI errors but also acquire the ability to devise appropriate corrective actions, thus honing their problem-solving skills in a practical context.

These AI literacy programs, an integral part of learner-centered EXPBME, can be thoughtfully designed to simulate a wide array of possible AI failures. By exposing healthcare professionals to various AI failure scenarios, these programs ensure that learners are well prepared to navigate the intricacies and challenges that may emerge in a technologically advanced healthcare environment. As AI technology continues its relentless advancement and becomes increasingly interwoven into the very fabric of healthcare delivery, the timeless learning theories that form the bedrock of medical education retain their crucial relevance.

Moreover, underscoring the complementary role of non-technical skills, such as effective communication, critical thinking, and ethical decision-making, becomes paramount in the context of learner-centered medical education within clinical settings. The integration of these non-technical skills is instrumental, not only to ensure that medical professionals are proficient in leveraging AI for optimal patient care but also to equip them with the essential skills necessary to navigate the complexities and uncertainties that accompany the integration of AI in modern healthcare. In other words, learner-centered EXPBME with sufficient reflection on experience can cultivate non-technical skill on controlling AI.

By embracing the principles of learner-centered EXPBME and prioritizing the holistic development of a well-rounded skill set, the medical community is poised to effectively harness the transformative potential of AI. Through this approach, the healthcare ecosystem can elevate patient care and safety to unparalleled heights, upholding the highest standards while embracing the technological wave of AI with a robust foundation of educational excellence.

## Conclusions

Despite rapid technological advancements, the fundamental experiential learning theory has shown resilience to changes. As we navigate the AI era, it becomes imperative to introduce an updated medical education curriculum that delves beyond the application and operation of AI, emphasizing a profound understanding of its principles and associated risks. While AI technology rapidly advances, the core learning theories remain steadfast. Therefore, an essential focus should be placed on recognizing the complementary function of non-technical skills in nurturing learner-centered EXPBME combined with AI literacy, accompanied by a commitment to continuous enhancement. Considering a learner-centered approach in EXPBME, integrating modules focused on professional development and career orientation into the medical curriculum can significantly improve students' preparedness in effectively planning their future careers. Learner-centered EXPBME, built upon these fundamental principles, acts as an essential resource to equip medical learners for the complex and constantly evolving role of AI in the field of medicine.
